# Robotic Flexible Ureteroscopy: A Step-by-Step Guide to the ILY® Platform

**DOI:** 10.1590/S1677-5538.IBJU.2026.0541

**Published:** 2026-08-13

**Authors:** Giovanni Scala Marchini, Gabriel K. Jahchan Alves, Rodrigo Javier Sánchez Sepúlveda, Alexandre Danilovic, Fábio Cesar Miranda Torricelli, Fábio Carvalho Vicentini, Carlos Alfredo Batagello, Rodrigo Perrella, Pedro Antonio Araújo Simões, Alexandre Gilberto Silva, William Carlos Nahas, Eduardo Mazzucchi

**Affiliations:** 1 Universidade de São Paulo Faculdade de Medicina Divisão de Urologia São Paulo Brasil Seção de Endourologia, Divisão de Urologia, Hospital das Clínicas, Faculdade de Medicina da Universidade de São Paulo, São Paulo, São Paulo, Brasil

## Abstract

**Introduction:**

Robotic flexible ureteroscopy (rfURS) is emerging as an alternative to manual flexible ureteroscopy for the treatment of upper urinary tract stones. Contemporary guidelines and reviews describe its potential ergonomic and radiation-related advantages, as well as comparable surgical outcomes (1-5). Initial clinical and simulation studies with the ILY® platform have demonstrated feasibility, safety, and a short learning curve, although standardized implementation remains limited (6-10). This video presents a step-by-step workflow for the safe and efficient use of the ILY® platform.

**Materials and Methods:**

A 43-year-old woman with a previous right double-J stent placed for obstructive pyelonephritis underwent rfURS for a 13-mm renal pelvic stone (1,497 HU; volume 1,214 mm³). The video demonstrates operating-room setup, robot calibration and sterile draping, robot positioning and fixation, docking to the ureteral access sheath and ureteroscope assembly, console-controlled navigation, laser lithotripsy, systematic renal inspection, and standardized undocking. A flexible suction sheath was used throughout the procedure.

**Results:**

Draping and docking times were 167 and 645 seconds, respectively. Robotic console time was 115 minutes, including 52 minutes of laser lithotripsy at 8 Hz and 1.2 J. Basket extraction was not required. Undocking was completed in 74 seconds, and total operative time was 130 minutes. No intraoperative complications or troubleshooting events occurred. Postoperative day-1 computed tomography confirmed stone-free status. The patient was discharged at 36 hours postoperatively without complications, as graded by the Clavien-Dindo classification (11). Surgeon satisfaction was rated 8 out of 10.

**Conclusion:**

rfURS using the ILY® platform was feasible, safe, and effective. A standardized step-by-step workflow may facilitate adoption, improve procedural consistency, and enhance surgeon ergonomics (12). rfURS represents a meaningful step toward autonomous surgery (13).

**Available at:**VIDEO

## Data Availability

Data will be available upon request.

## References

[1] European Association of Urology (2025). EAU Guidelines on Urolithiasis. Presented at the EAU Annual Congress Madrid 2025. [Internet].

[2] Gauhar V, Traxer O, Cho SY, Teoh JYC, Sierra A, Gauhar V (2022). Robotic retrograde intrarenal surgery: a journey from "back to the future". J Clin Med.

[3] Ergül RB, Özervarli MF, Panthier F, Nedbal C, Tsaturyan A, Pietropaolo A (2025). Surgical outcomes of robotic surgery for kidney stones: a systematic review and meta-analysis from Section of YAU and EAU Endourology. World J Urol.

[4] Kim HJ, Elises JCR, Castillo CJ, Aljameel N, Alzahrani F, Sulaiman O (2025). Robot-assisted retrograde intrarenal surgery: a mini review of the literature on robotic surgery platforms for endoscopic stone surgery. Eur Urol Focus.

[5] Davis NF, Quinlan MR, Poyet C, Lewis S, D’Costa MR, Wollin DA (2023). Comparison of treatment outcomes for fluoroscopic and fluoroscopy-free endourological procedures: a systematic review on behalf of the European Association of Urology Urolithiasis Guidelines Panel. Eur Urol Focus.

[6] Fiori C, Cossu M, Poggio M, Quarà A, Di Dio M, De Luca S (2023). Initial experience with robot-assisted ureteroscopy with ILY® robot. Minerva Urol Nephrol.

[7] Łaszkiewicz J, Krajewski W, Nowak Ł, Chorbińska J, Del Giudice F, Sójka A (2024). Retrograde intrarenal surgery using the ILY robotic flexible ureteroscope: a single-centre experience. Cent European J Urol.

[8] El-Hajj A, Abou Chawareb E, Zein M, Wahoud N (2024). First prospective clinical assessment of the ILY® robotic flexible ureteroscopy platform. World J Urol.

[9] Farré A, Angerri O, Kanashiro A, Casadevall M, Uleri A, Balañà J (2024). Robot-assisted retrograde intrarenal surgery: first clinical experience with the ILY® system. BJU Int.

[10] Alanazi A, Madden A, Vanpoperinghe L, Calugaru P, Quarà A, Dokter L (2024). A bench-top evaluation of the ILY® robotics-assisted teleconsole system in ureteroscopy by medical students, residents and urologists: does prior videogaming experience help shape the learning curve?. World J Urol.

[11] Dindo D, Demartines N, Clavien PA (2004). Classification of surgical complications: a new proposal with evaluation in a cohort of 6336 patients and results of a survey. Ann Surg.

[12] Almeida GL, Busato WFS, Berger AK, Codagnone V, Winter G, Kinchescki R (2025). First Brazilian series of urologic surgeries using Versius robotic multiport platform. Int Braz J Urol.

[13] Andras I, Piramide F, Bravi CA, Di Maida F, Turri F, Lambert E (2025). Systematic review and clinical outcomes of new robotic systems in urology. Int Braz J Urol.

